# Perilipin-2 promotes lipid droplet-plasma membrane interactions that facilitate apocrine lipid secretion in secretory epithelial cells of the mouse mammary gland

**DOI:** 10.3389/fcell.2022.958566

**Published:** 2022-09-09

**Authors:** Jenifer Monks, David J. Orlicky, Andrew E. Libby, Monica Dzieciatkowska, Mark S. Ladinsky, James L. McManaman

**Affiliations:** ^1^ Division of Reproductive Sciences, University of Colorado Anschutz Medical Campus, Aurora, CO, United States; ^2^ Graduate Program in Integrated Physiology, University of Colorado Anschutz Medical Campus, Aurora, CO, United States; ^3^ Department of Pathology, University of Colorado Anschutz Medical Campus, Aurora, CO, United States; ^4^ Department of Biochemistry and Molecular Genetics, University of Colorado Anschutz Medical Campus, Aurora, CO, United States; ^5^ Division of Biology and Biological Engineering, California Institute of Technology, Pasadena, CA, United States

**Keywords:** apocrine secretion, lipid droplet, mammary gland, molecular interaction, perilipin 2/adipophilin, plasma membrane, secretory epithelium

## Abstract

Secretory epithelial cells (sMEC) in mammary glands of lactating animals secrete lipids by a novel apocrine mechanism in which cytoplasmic lipid droplets (LD) contact and are enveloped by elements of the apical plasma membrane (APM) before being released into the lumen of the gland as membrane bound structures. The molecular properties of LD-APM contacts and the mechanisms regulating LD membrane envelopment and secretion are not fully understood. Perilipin-2 (Plin2) is a constitutive LD protein that has been proposed to tether LD to the APM through formation of a complex with the transmembrane protein, butyrophilin1a1 (BTN) and the redox enzyme, xanthine oxidoreductase (XOR). Using mice lacking Plin2 and physiological inhibition of apocrine lipid secretion, we demonstrate that LD-APM contact and envelopment are mechanistically distinct steps that they are differentially regulated by Plin2 and independent of LD secretion. We find that Plin2 is not required for formation of LD-APM contacts. However, it increases the percentage of LD that contact the APM and mediates enlargement of the LD-APM contact zone as LD undergo membrane envelopment. The effects of Plin2 LD-APM interactions are associated with increased abundances of BTN, XOR and Cidea, which are implicated as mediators of LD-APM contact formation, on membranes surrounding secreted LD, and with promotion of glycocalyx remodeling at LD-APM contact sites. We propose that Plin2 does not directly mediate contact between LD and the APM but acts by enhancing molecular interactions that stabilize LD-APM contacts and govern membrane envelopment of LD during apocrine lipid secretion. Plin2 does not appear to significantly affect the lipid content of milk in fully lactating animals, but it does increase lipid secretion at the onset of lactation in primaparous dams, which suggest a role in facilitating apocrine lipid secretion in sMEC during their initial transition to a secretory phenotype.

## 1 Introduction

Lipid droplets are ubiquitous organelles that function as regulators of cellular energy homeostasis in plant and animal cells through effects on lipid storage, trafficking and metabolism (Murphy 2001). In secretory epithelial cells of the mammary gland (sMEC), LD also are secreted into milk where they serve the primary source of neonatal calories (Oftedal 1984). LD secretion occurs by an apocrine mechanism in which LD are enveloped and secreted as intact, membrane encapsulated, structures known as milk fat globules (MFG), in response to oxytocin stimulated contraction of myoepithelial cells (Patton and Keenan 1975; Mather, et al., 2019). This method of lipid secretion differs from vesicle-mediated exocytic mechanisms used to secrete serum lipids (Vance and Vance 1990) and from holocrine mechanisms of lipid secretion used by other exocrine organs (Wooding 1980).

A distinctive feature of apocrine LD secretion by sMEC is the formation of stable, electron-dense 10–20 nm contacts between the LD and the apical plasma membrane (APM) (Wooding 1971) that induce alterations in the plasma membrane structure and spatial remodeling of integral membrane proteins and the glycocalyx (Peixoto de Menezes and Pinto da Silva 1978; Mather and Keenan 1998; Monks, et al., 2016), which are proposed to facilitate the apocrine secretion process (Vorbach, et al., 2002; Ogg, et al., 2004; Monks, et al., 2016). However, significant gaps remain in our understanding of the molecular and structural features that define LD-APM contacts, the mechanisms governing their formation and stabilization, and the functional relationships between membrane contact, envelopment, and secretion of LD (Monks, et al., 2020).

LD-APM contacts are hypothesized to be mediated by a tethering complex composed of the integral plasma membrane protein butyrophilin1n1 (BTN1a1/BTN), the redox enzyme xanthine oxidoreductase (XDH/XOR), and the constitutively-associated LD coat protein, perilipin-2 (Plin2) (Mather and Keenan 1998; McManaman, et al., 2002; Heid and Keenan 2005). These proteins co-localize at LD-APM contact sites and can be isolated from membranes surrounding MFG as a detergent resistant complex (McManaman, et al., 2002). Gene deletion studies demonstrated that BTN and XOR are required for formation of LD-APM contacts in sMEC of lactating mice and that their absence interferes with apocrine lipid secretion and lactation (Vorbach, et al., 2002; Ogg, et al., 2004; Monks, et al., 2016). Plin2 deletion or disruption of Plin2 function are associated with lactation failure in primiparous dams (McManaman, et al., 2013) (Russell, et al., 2011) and viral expression of mutant forms of Plin2 is linked to impaired apocrine lipid secretion in WT dams (Chong, et al., 2011). However, the direct involvement of Plin2 in formation of LD-APM contacts or in apocrine secretion of lipids has not been demonstrated.

To test the hypothesis that Plin2 mediates LD-APM contacts and apocrine lipid secretion in sMECs, we investigated the effects of Plin2 deletion on the formation and molecular properties of LD-APM contacts and apocrine lipid secretion using lactating multiparous Plin2-Null and WT dams, which have similar litter growth rates (McManaman, et al., 2013), in lactating dams in which apocrine lipid secretion was physiologically inhibited by preventing nursing-stimulated oxytocin release (Mather, et al., 2019), and in primiparous WT and Plin2-Null dams during the initial onset of lactation. Our data provide evidence that apocrine lipid secretion involves molecular and physiologically distinct mechanisms that regulate membrane contact, envelopment, and secretion of LD. We find that formation of LD-APM contacts does not require Plin2. However, Plin2 actions enhance the number and size of LD-APM contacts, promote apical membrane envelopment of LD, and facilitate reorganization of membrane properties at LD-APM contact sites, and increase LD secretion at the initial onset of lactation.

## 2 Methods

### 2.1 Animals

Wild type and Plin2-Null females were on the C57Bl/6J background and obtained from breeding colonies maintained in the AAALAC-accredited Center for Comparative Medicine at the University of Colorado Anschutz Medical Campus (CUAMC) as previously described (McManaman, et al., 2013). Pregnant and lactating females were housed at 22°C in microisolator cages equipped with automated air and water on a 10:14 h dark:light cycle with *ad libitum* access to food. All animals were provided with cotton nesting material enrichment, and breeders and experimental females were additionally provided with shredded paper to construct an enclosed nest. Pregnant females were housed individually prior to parturition. The day a litter was first seen was counted as lactation day 1 (L1). Litters were standardized to five pups per dam by culling. Culled neonatal animals were humanely euthanized using CO_2_ followed by decapitation. Studies were performed on mammary tissue and milk samples from dams at L10. Mammary tissue was removed from dams euthanized by CO_2_ followed by cervical dislocation by personnel trained in their care. Pups from experimental dams were euthanized by CO_2_ followed by cervical dislocation. All animal experiments and procedures were approved by the University of Colorado School of Medicine’s Institutional Animal Care and Use Committee on protocol 00985.

### 2.2 Milk collection and milk fat globule membrane isolation

Pups were removed from dams on L10 for 3 h to allow milk accumulation in mammary glands. Milk removal was essentially as described (DePeters and Hovey 2009) except that xylazine alone was used as a sedative and muscle relaxant. Briefly, xylazine was given I.P. at a dose of 8 mg/kg. When the mouse was relaxed enough to have ceased ambulation around the cage (about 5 min), the milking procedure was initiated. The mouse was picked up, and with gentle hand-restraint, a single dose of oxytocin (0.5 IU, 0.25 ml in sterile saline) was given I.P. Milk let-down occurred within 1 min and milk removal was started. Our standard milking apparatus, attached to house vacuum, was used. Hand restraint was used throughout the milking procedure. Milk was collected and processed at room temperature to avoid changes in protein segregation between phases (Dickow, et al., 2011).

Milk fat globules were isolated by mixing fresh whole milk 1:1 with 10% sucrose and layered under 10 + ml of PBS. This preparation was centrifuged at × 1,500 g at room temperature. The floated globules were collected, washed twice with PBS and stored at −80°C. Frozen MFG were thawed on ice and subjected to Dounce homogenization- 100 strokes, in ice water bath to prevent warming. Resulting liquid, and one PBS wash of the homogenizer, were combined and centrifuged at × 22,000 g for 20 min at 4°C. The resulting membrane pellet was stored at −80°C until processing for proteomics.

### 2.3 Proteomic analysis

MFGM preparations from 10 WT to 15 Plin2-Null dams were pooled into 2 and 3 samples respectively and processed for proteomic analysis essentially as described (Wisniewski, et al., 2009). Proteins were digested according to the FASP protocol using a 30 kDa molecular weight cutoff filter. In brief, samples were mixed in the filter unit within 8 M urea in 0.1 M Tris-HCl, pH 8.5, and centrifuged at 14,000 g for C for 15 min. The proteins were reduced by addition of 100 μL of 10 mM DTT in 8 M urea in 0.1 M Tris-HCl, pH 8.5, with incubation for 30 min at room temperature and the device was centrifuged. Subsequently, 100 μL of 55 mM iodoacetamide in 8 M urea in 0.1 M Tris-HCl, pH 8.5, was added to the samples, with incubation for 30 min at room temperature in the dark followed by centrifugation. Afterward, three washing steps with 100 μL of 8 M urea in 0.1 M Tris-HCl, pH 8.5, solution were performed, followed by three washing steps with 100 μL of 50 mM ABC buffer. Proteins were digested with trypsin overnight at 37°C. Peptides were recovered from the filter using 30% acetonitrile. The volume of the eluted sample was reduced to 2 μL in a vacuum centrifuge and reconstituted to 50 μL with 0.1% formic acid.

### 2.4 LC/MS/MS analysis

Samples were analyzed on an LTQ Orbitrap Velos mass spectrometer (Thermo Fisher Scientific) coupled to an Eksigent nanoLC-2D system through a nanoelectrospray LC−MS interface. A sample volume of 8 μL was injected into a 10 μL loop using the autosampler. To desalt the sample, material was flushed out of the loop and loaded onto a trapping column (ZORBAX 300SB-C18, dimensions 5 × 0.3 mm, 5 μm) and washed with 0.1% formic acid at a flow rate of 5 μL min−1 for 5 min. The analytical column was then switched on-line at 600 nL min−1 over an in-house 100 μm i.d. X 200 mm fused silica capillary packed with 4 μm 80A° Synergi Hydro C18 resin (Phenomex; Torrance, CA, United States). After 10 min of sample loading, the flow rate was adjusted to 350 nL min−1, and each sample was run on a 120 min linear gradient of 5–40% acetonitrile with 0.1% formic acid to separate the peptides. LC mobile phase solvents and sample dilutions used 0.1% formic acid in water (Buffer A) and 0.1% formic acid in acetonitrile (Buffer B) (Chromasolv LC–MS grade; Sigma-Aldrich, St. Louis, MO, United States). Data acquisition was performed using the instrument-supplied Xcalibur (version 2.1) software. The mass spectrometer was operated in positive ion mode. Each survey scan of m/z 400–2000 was followed by collision-assisted dissociation (CAD) MS/MS of 20 most intense precursor ions. Singly charged ions were excluded from CAD selection. Normalized collision energies were employed using helium as the collision gas. Each sample was analyzed in duplicate.

### 2.5 Database searching and protein identification

MS/MS spectra were extracted from raw data files and converted into mgf files using a PAVA script (UCSF, MSF, San Francisco, CA, United States). These mgf files were then independently searched against the mouse SwissProt database using an in-house Mascot server (Version 2.5, Matrix Science, London, United Kingdom). Mass tolerances were ±15 p.p.m. for MS peaks, and ±0.6 Da for MS/MS fragment ions. Trypsin specificity was used allowing for one missed cleavage. Met oxidation, protein N-terminal acetylation, peptide N-terminal pyroglutamic acid formation, deamidation of asparagine, glutamine and tryptophan, sulphone of methionine, and tryptophan oxidation to formylkynurenin of tryptophan were allowed for variable modifications while carbamidomethyl of Cys was set as a fixed modification. The MS proteomics data have been deposited to the ProteomeXchange Consortium via the PRIDE partner repository with the dataset identifier. Scaffold (version 5.0.0, Proteome Software, Portland, OR, United States) was used to validate MS/MS-based peptide and protein identifications. Peptide identifications were accepted if they could be established at greater than 95.0% probability as specified by the Peptide Prophet algorithm. Protein identifications were accepted if they could be established at greater than 99.0% probability and contained at least five identified unique peptides. Protein spectral counts were converted to normalized spectral abundance factors (NSAF) (Zybailov, et al., 2006) in Scaffold for quantitation. NSAF values were analyzed in GraphPad Prism (9.3.1) by unpaired *t*-test using the two-stage step-up false discovery method of Benjimani, Krieger and Yekutieli to correct for multiple comparisons. A q value of 0.01 was considered significant.

### 2.6 Lipidomic analysis

Milk lipids were analyzed by GC-MS/MS according to published procedures (Libby, et al., 2016). Frozen milk was mixed with Folch reagent (2:1 CHCl_3_:MeOH) containing 300 μg of tritridecanoin reference standard (Nu-Check Prep Inc., Elysian, MN). Homogenates were diluted further with Folch reagent, treated with 800 μL of 0.9% sodium chloride solution, vortexed and centrifuged at 4000 RPM for 5 min. The organic phase was removed and dried under N_2_ gas. Total lipids were resuspended in 330 μL 100% chloroform and applied to HyperSep SI SPE columns (Thermo Scientific, Waltham, MA) pre-equilibrated with 15 column volumes chloroform. Neutral lipids were eluted with a total of 3 ml chloroform, dried under N_2_, and resuspended in 1 ml methanol containing 2.5% H_2_SO_4_. Fatty acid methyl ester (FAME) production was initiated by heating at 80°C for 1.5 h 1 ml of HPLC-grade water was added to quench the reactions, and FAMEs/cholesterol was extracted with 200 uL hexane. A Trace 1310 GC with a TG-5MS column (Thermo Scientific, Waltham, MA) was used to separate lipids chromatographically, and lipids were analyzed with an ISQ single quadrapole mass spectrometer. Xcalibur software (Thermo Scientific) was used to calculate peak areas. Areas were normalized to the tritridecanoin reference standard and then to milk volume.

### 2.7 Imaging

#### 2.7.1 Electron microscopy and tomography

Mammary tissue was removed from dams on L10 following litter separation for 2 h. After euthanasia, mammary glands were removed and pre-fixed in 3% glutaraldehyde, 1% paraformaldehyde, 5% sucrose in 0.1M sodium cacodylate pH 7.2 (Electron Microscopy Sciences (EMS), Hatfield, PA) at 4°. Samples were dissected into ∼0.25 mm^3^ pieces, placed into brass planchettes (Ted Pella, Inc.) and ultra-rapidly frozen with a HPM-010 High Pressure Freezing Machine (Bal-Tec/ABRA). Vitrified samples were transferred under liquid nitrogen to cryotubes (Nunc) prefilled with 2.5% OsO_4_, 0.05% uranyl acetate in acetone. Samples were placed in an AFS-2 Freeze-Substitution Machine (Leica Microsystems, Vienna) and processed at −90°C for 72 h; warmed to −20°C over 24 h, then held at −20°C for 12 h. Samples were brought to room temperature, rinsed 3x with acetone and then infiltrated into Epon-Araldite resin (EMS). Tissue samples were flat-embedded between two Teflon-coated glass microscope slides and the resin polymerized at 60°C for 24 h. Flat-embedded samples were observed with a phase-contrast light microscope to select suitable regions for EM study. These regions were excised with a microsurgical scalpel and glued to the tips of plastic sectioning stubs. Serial semi-thick (150–170 nm) sections were cut with a UC6 ultramicrotome (Leica Microsystems) using a diamond knife (Diatome, Ltd., Switzerland) and collected onto formvar-coated copper-rhodium 1 mm slot grids (EMS). Sections were stained with 3% uranyl acetate and lead citrate and 10 nm colloidal gold particles were placed on both surfaces of the grid for use as fiducial markers for tomographic image alignment. Sections were imaged with a Tecnai T12 transmission electron microscope (120 keV, Thermo-Fisher Scientific) using a Dual-Axis Tomography sample holder (Model 2040; E.A. Fischione Instruments, Inc. Export, PA.). For dual-axis tomographic analysis, grids were tilted+/−62° and images taken at 1° intervals. The grid was then rotated 90° and a second tilt-series was acquired about the orthogonal axis. Data was acquired automatically using the SerialEM software package (Mastronarde 2005)**.** Tomograms were calculated, analyzed, and modeled using the IMOD software package (Kremer, et al., 1996) on iMac Pro and M1 computers (Apple Inc.).

#### 2.7.2 Immunohistochemistry

Formaldehyde-fixed, paraffin-embedded tissue was sectioned into 5 μm slices and stained with hematoxylin and eosin (H&E). Histological images were captured on an Olympus BX51 microscope equipped with a 4 Mpixel Macrofire digital camera (Optronics, Goleta, CA, United States) using the PictureFrame Application 2.3 (Optronics). For immunostaining, sections were deparaffined with xylene and graded ethanols and antigen retrieval was performed using a citrate-based solution (VectorLabs, Burlingame, CA, United States). The sections were permeabilized with TX100 and glycine and blocked with 10% donkey serum with saponin, and immunostained with antibodies with validated specificity: rabbit anti-Plin2 (1:100) (Russell, et al., 2007), or guinea pig anti-Plin2 (1:200, 2R-AP002, Fitzgerald, Acton, MA, United States), rabbit anti-Plin3 (TIP47, 1:100) (Russell, et al., 2007), or guinea pig anti-Plin3 (1:200, 20R-2602, Fitzgerald, Acton, MA, United States), guinea pig anti-butyrophilin (1:200, GP153, Progen Biotechnik GmbH, Heidelberg, Germany), rabbit anti-XOR (1:100, ab133268, EPR4606, Abcam, Waltham Mass. United States) (McManaman, et al., 2002), rabbit anti-Cidea (1:100, aa200-214, AbD Serotec/Bio-Rad, Raleigh, NC, United States), rabbit anti-Rab18 (1:200, 11304-1-AP) Proteintech, Rosemont Ill United States or (1:100, ab119900 Abcam, Waltham Mass. United States), wheat germ agglutinin (Alexa 633 conjugate, Invitrogen, Carlsbad, CA, United States), and DAPI (Sigma, D9542). Secondary antibodies were from Jackson ImmunoResearch (West Grove, PA, United States). Sections were mounted with ProLong Gold (Molecular Probes, Eugene, OR, United States). Imaging was performed on a Marianas Spinning Disc confocal microscope (Intelligent Imaging Innovations, Inc., Denver, CO, United States), using a ×100 objective (plan apochromat, NA 1.4). Analysis was performed using SlideBook v. 6.0 (Intelligent Imaging Innovations), on 10–20 images from at least three animals per group.

#### 2.7.3 Statistical analyses

Statistical analyses were performed using GraphPad Prism (9.3.1) programs. Unless otherwise indicated, *p*-values < 0.05 were considered significant.

## 3 Results

### 3.1 LD-APM contacts in sMEC

**FIGURE 1:**
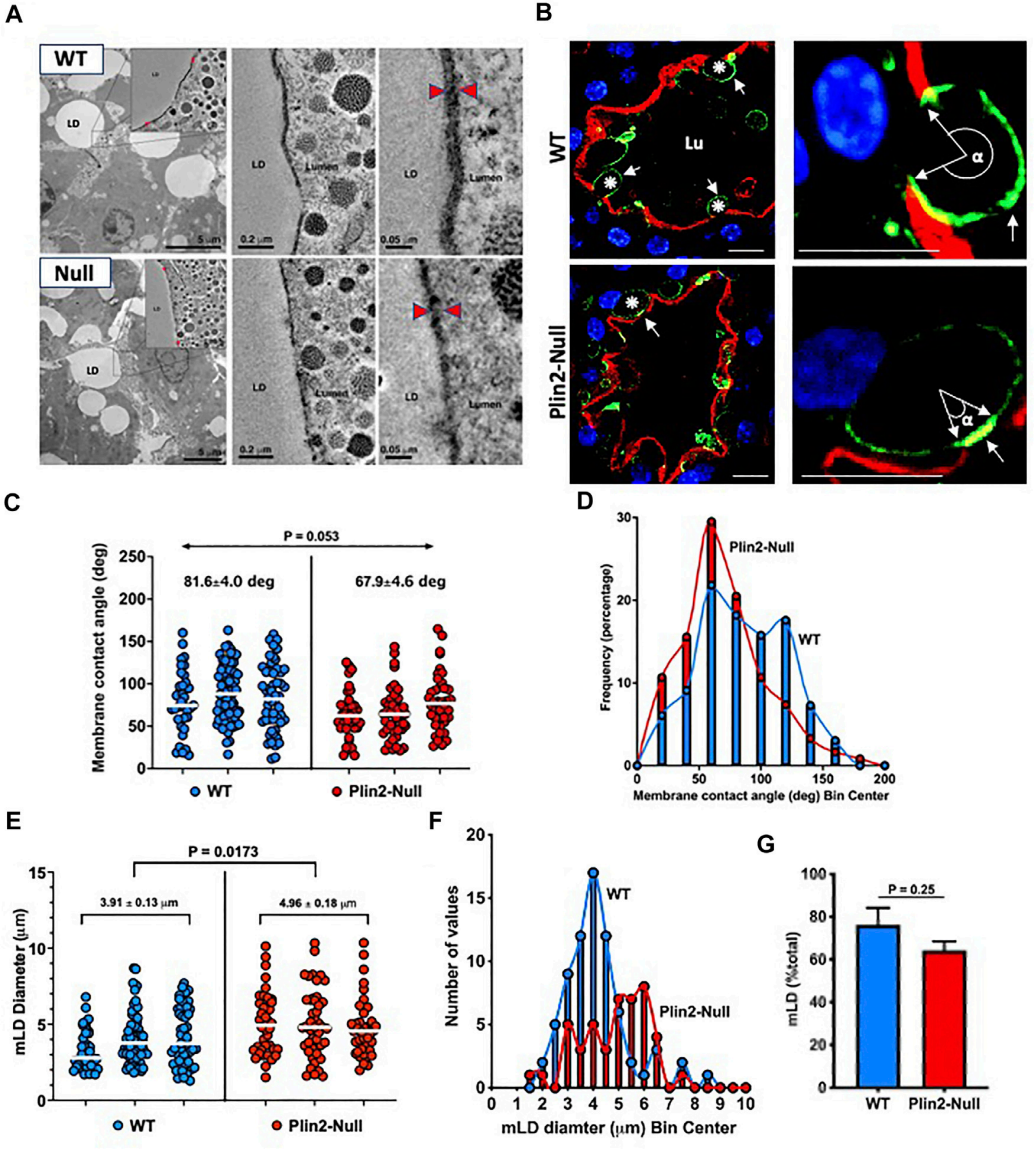
LD-APM contacts. **(A)** Electron tomograms from mammary glands of WT and Plin2-Null dams at L10. Left panels show lower magnification images of mammary alveoli, the box outlines contact between a LD and the apical membrane that is further magnified in insets and subsequent images in the middle and right panels. Red arrowheads in the right panels indicate LD-APM contact zones. Scale bars are indicated in each panel. **(B)** Mammary alveoli from WT and Plin2-Null mammary glands at L10 immunostained for Cidea (green) to identify LD and with Alexa594-WGA (red) to identify the apical plasma membrane (APM). Left panels show LD contacting the APM (asterisks). LD-APM contact sites are identified by Cidea concentration at the APM (white arrows). Right panels are higher magnification images of LD-APM contacts (white arrows) in WT and Plin2-Null sMEC showing membrane contact angles (α). Scale bars = 10 µm. **(C)** LD-APM contact angles quantified in sMEC from 20–40 randomly selected mammary alveoli from 3 WT (blue) and Plin2-Null (red) dams. Horizontal bars indicate median membrane contact angles. Average median membrane contact angles ±SEM are shown above each group. *p* value for group differences determined by nested *t* test is shown at the top of figure. **(D)** Histogram showing size distributions of membrane contact angles for each genotype. **(E)** Diameters of LD contacting the APM (mLD) in sMEC from 20–40 randomly selected mammary alveoli from 3 WT and Plin2-Null dams. Horizontal bars indicate median diameters. Average median mLD diameters ±SEM are shown above each group. *p*-values for group differences were determined by nested *t*-test and are shown at the top of figure. **(F)** Histogram showing the mLD size distribution for each genotype. **(G)** Percentage of LD contacting the APM. Values are averages ±SEM from sMEC in 20–40 randomly selected alveoli from 3 WT and Plin2-Null dams. *p*-values were determined by Students t-test.

We investigated the effects of Plin2 on LD-APM contacts in sMEC from multiparous WT and Plin2-Null dams on lactation day 10. EM tomography demonstrates extensive electron dense zones of contact between LD and the APM in both genotypes (Figure 1A), which suggest that Plin2 actions are not essential for stable LD-APM contacts to occur. However, the width of the LD-APM interface within the contact zone in WT sMEC (∼22 nm) appears to be larger than that in Plin2-Null cells (∼14 nm), suggesting that Plin2 may modulate LD-APM interactions at contact sites. We investigated this possibility by quantifying the physical dimensions of LD-APM contacts in sMEC by confocal immunofluorescence imaging of cell death-inducing DFFA-like effector A (Cidea), which is enriched at LD-APM contact sites in mouse sMEC during apocrine lipid secretion (Monks, et al., 2016; Monks, et al., 2020). Cidea immunofluorescence is enriched at LD-APM contacts in sMEC of both WT and Plin2-Null dams (Figure 1B). However, the size of the Cidea-enriched contact zone appears smaller and fewer LD appeared to contact the APM in Plin2-Null sMEC. We used the angle formed between the LD center and the margins of Cidea immunofluorescence within the contact zone, which we denote as α and refer to as the membrane contact angle (MCA), to estimate LD-APM contact zone size (Figure 1C). The average median MCA in Plin2-Null sMec (67.90) is approximately 17% less than that of WT sMEC (81.60) (*p* = 0.052). Histogram analysis of MCA distribution (Figure 1D) in WT cells showed two peaks centered at 60 and 120°, whereas a single peak centered at 60° was present in Plin2-Null cells, which indicate that loss of Plin2 impairs expansion of LD-APM contacts during apocrine lipid secretion.

We also find that the Plin2 deletion influences the size of membrane-bound lipid droplets (mLD) (Figure 1E). The average mLD median diameter in sMEC from WT dams (3.91 ± 0.13 µm) is significantly less that in sMEC from Plin2-Null dams (4.96 ± 0.18 µm; *p* = 0.0173). Analysis of mLD size distribution (Figure 1F) shows that mLD diameters have a unimodal distribution peaking at approximately 4 µm in WT sMEC, whereas in Plin2-Null cells mLD diameters have a multimodal distribution with peaks at 3, 4 and 6 µm. We did not find a significant effect of Plin2 deletion on the percentage of LD in contact with the APM in lactating animals, although the percentage trended lower in Plin2-Nulls (Figure 1G). Together these data indicate that Plin2 actions influence processes controlling the size of LD forming contacts with the APM as well as the extent LD-APM contact.

### 3.2 LD-APM responses to inhibiting apocrine lipid secretion

**FIGURE 2:**
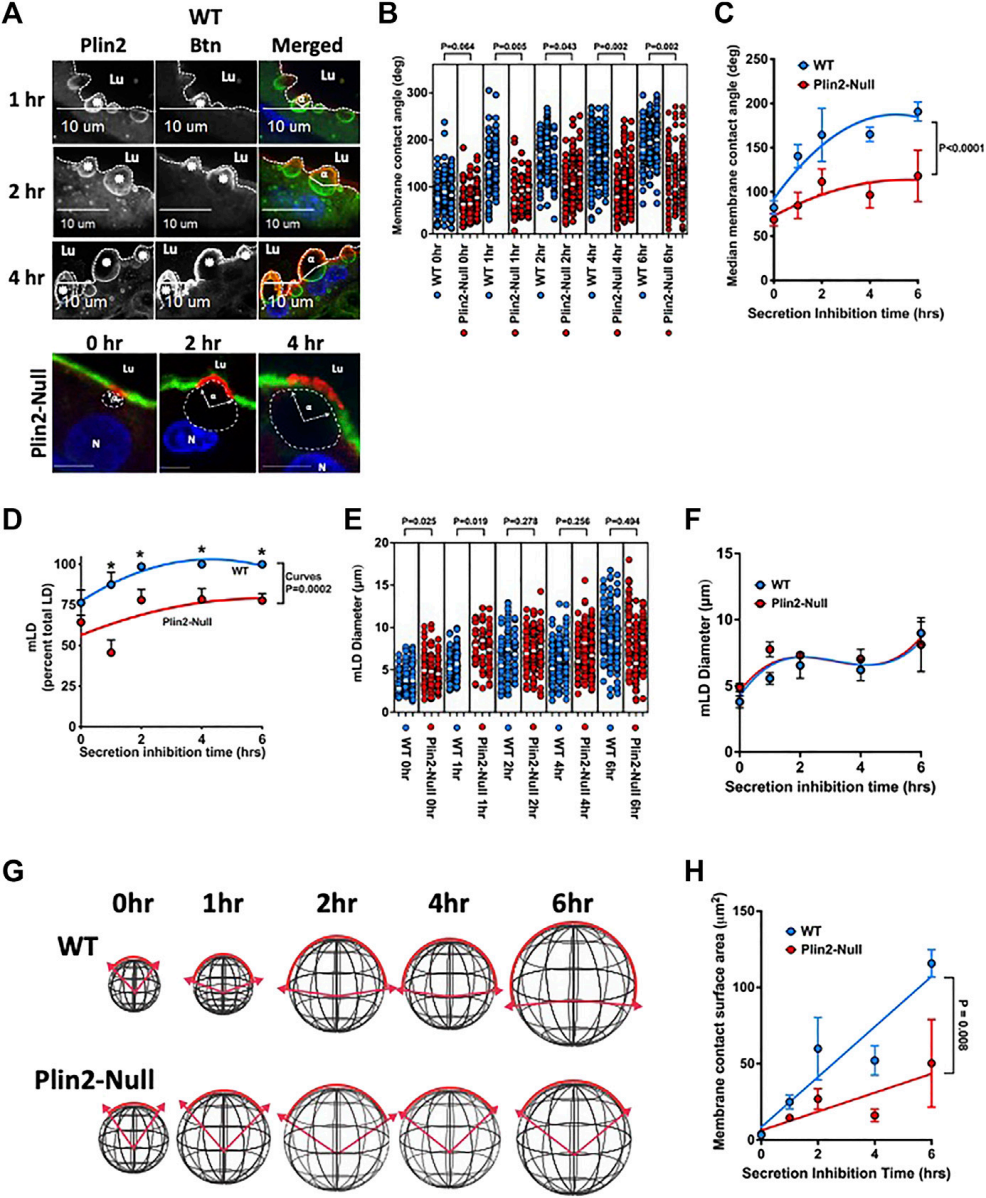
LD-APM envelopment. **(A)** Immunofluorescence images showing time-dependent expansion of LD-APM contacts following the inhibition of apocrine lipid secretion in sMEC from WT and Plin2-NULL mammary glands. Sections from WT cells immunostained for Plin2 (green in merged panels) and BTN (red in merged panels) to identify LD (asterisks) and LD-APM contacts respectively. LD-APM contact angles (α) are indicated in merged panels. Apical borders are indicated by the dotted line, luminal space is labeled (Lu) and scale bars are indicated. Sections from Plin2-Null glands were stained for BTN (red) and Alexa488-labled WGA to identify LD-APM contact sites and apical borders respectively. LD in these images were localized by autofluorescence and outlined by dashed lines. LD-APM contact angles (α), luminal space (Lu) and nuclei (N) are labeled. Scale bars are 10 µm. **(B)** Changes in LD-APM contact angles in WT and Plin2-Null sMEC following secretion inhibition. LD-APM contact angles were quantified in sMEC from 20–40 randomly selected mammary alveoli from 3 WT and Plin2-Null dams at each time point, except 1h in which measurements were from 2 WT to 3 Plin2-Null dams. White bars indicate median membrane contact angles. *p* values for group differences were determined by nested *t*-test and are shown above each comparison. **(C)** Curves describing changes in median LD-APM contact angles following inhibition were generated using centered second order polynomial least-squares-fit (Prism 9.3.1). Values are means ± SEM (N = 3, 60–80 alveoli/dam). *p* value corresponds to differences between curves determined by extra sum-of-squares F test. **(D)** The percentage of LD contacting the APM (mLD) following secretion inhibition in WT and Plin2-Null dams. Curves were generated using centered second order polynomial least-squares-fit (Prism 9.3.1). Values are averages ±SEM (N = 3, 60–80 alveoli/dam). *p* value corresponds to differences between curves determined by extra sum-of-squares F test. **(E)** Effects of inhibiting secretion on mLD diameters from WT and Plin2-Null dams. mLD diameters were quantified in sMEC from 20–40 randomly selected mammary alveoli from 3 WT and Plin2-Null dams at each time point. White bars indicate median values. *p* values for group differences at each time point were determined by nested *t*-test and are shown above each comparison. **(F)** Curves describing effects of secretion inhibition time on median diameters of mLD were generated using centered third order polynomial least-squares-fit (Prism 9.3.1). Values are averages ±SEM (N = 3, 60–80 alveoli/dam). **(G)** Diagrams depicting how measured changes in LD diameters and LD-APM contact angles are projected to affect the extent LD-APM engagement in WT and Plin2-Null dams at 0, 1, 2, 4, or 6 h after inhibiting secretion. **(H)** Change in calculated membrane area contacting LD in WT and Plin2-Null dams as a function of time after inhibiting secretion. Curves were generated using linear least-squares-fit. *p*-values refer to differences in the slopes of the curves determined by extra sum-of-squares F test.

The presence of significant differences in sMEC LD content and LD-APM contacts in actively secreting mammary glands (Mather, et al., 2019) potentially increase variability in the properties of LD-APM contacts. To better understand how Plin2 affects LD-APM interactions, we inhibit oxytocin-dependent apocrine lipid secretion by removing litters from WT and Plin2-Null dams (Masedunskas, et al., 2017). Using the enrichment of BTN on the APM to define LD-APM contacts (Monks, et al., 2016) (Figure 2A), we find that MCA increases progressively with time after inhibiting apocrine lipid secretion in both sMEC of both WT (*p* < 0.001) and Plin2-Null (*p* = 0.01) glands (Figures 2B,C), demonstrating that APM envelopment of LD is independent of their secretion. However, MCA values in Plin2-Null cells are significantly less than those in WT cells throughout the period of inhibition (Figure 2C). In WT cells, the average median MCA increases from 82° prior to inhibiting secretion to a maximum of 190° 6 h after inhibition. Whereas in Plin2-Null cells, MCA increases from 62° prior to inhibiting secretion to a maximum of 118° 6 h after inhibition (Figure 2C). The curves describing changes in MCA values with time of inhibition differed significantly between WT and Plin2-Null cells (*p* < 0.001).

We also find that Plin2 influences the percentage and size of LD contacting the APM in WT and Plin2-Null sMEC. The percentage of LD contacting the APM in WT cell increased from 76% prior to inhibition to 100% 2 h after inhibition (*p* = 0.032) (Figure 2D). In sMEC from Plin2-Null dams, the percentage of LD in contact with the APM increased from 64% prior to inhibiting secretion to maximum of 78% from 4 to 6 h after inhibition. The percentage of LD contacting the APM in sMEC from WT dams were significantly greater than that in cells from Plin2-Null dams at 1,2,4 and 6 h after initiating inhibition, and the curves describing the effects of time on changes in the percentage of LD-APM contacts in WT and Plin2-Null cells differed significantly (*p* = 0.0002) (Figure 2D).

Inhibiting apocrine lipid secretion also increases mLD diameters in WT (*p* = 0.0002) and Plin2-Null (0.028) sMEC (Figure 2E). However, other than the 0 and 1 h values we did not detect differences in mLD diameters between WT and Plin2-Null cells (Figure 2E), and the curves describing time dependence of inhibition on mLD diameters were identical for cells from both genotypes (Figure 2F). Using mLD diameter and MCA measurements, we estimated the effects of inhibiting apocrine lipid secretion on the surface areas of LD-APM contacts in WT and Plin2-Null sMEC. Membrane surface areas were calculated using the formula for the surface area of an arc segment, Area = 2πR^2^(1−cos θ). Arc segments corresponding to mLD diameters and MCA measurements in WT and Plin2-Null sMEC cells following the inhibition of secretion are depicted diagrammatically in Figure 2G. Changes in calculated membrane surface areas of LD-APM contacts are shown as a function of time after secretion inhibition in Figure 2H. The results show that membrane surface areas engaged in LD-APM increase linearly following inhibition of secretion in WT and Plin2-Null sMEC. However, the rate of increase in WT dams is more than twice that of Plin2-Null dams (*p* = 0.008).

### 3.3 Apical membrane protein properties

**FIGURE 3:**
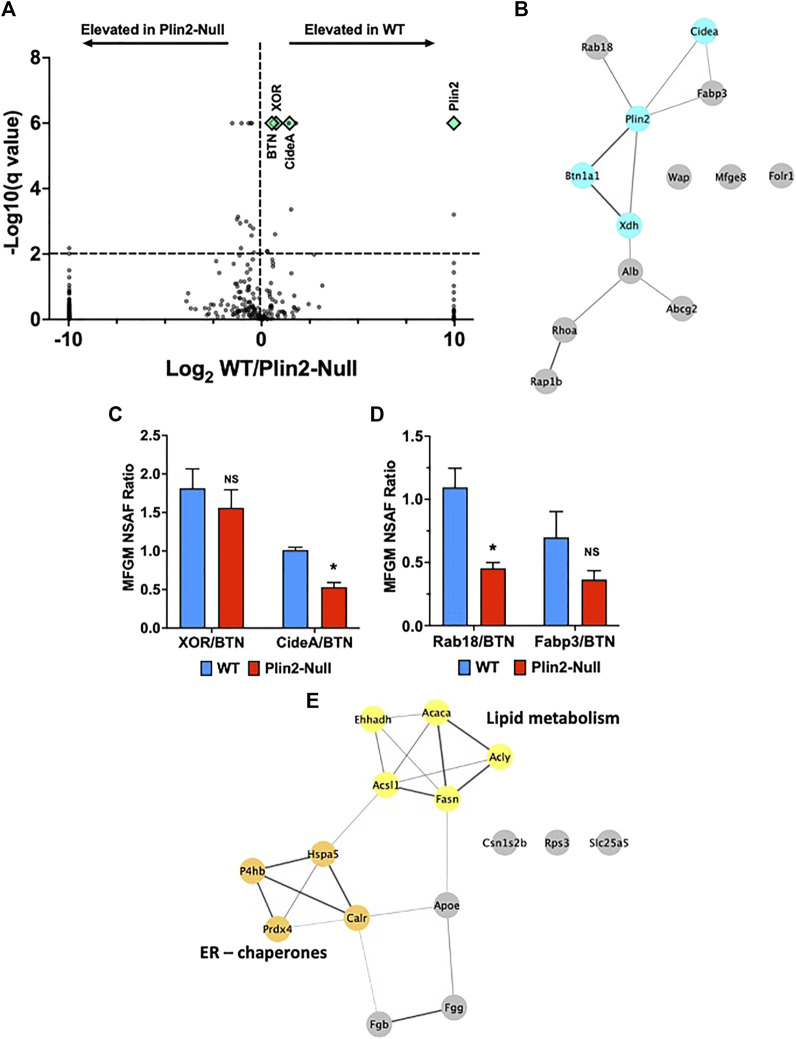
Milk fat globule membrane (MFGM) protein composition. **(A)** Volcano plots showing log_2_ fold change in normalized spectral abundance factor (NSAF) ratios for proteins in WT and Plin2-Null MFGM versus −log_10_ of their q-values, multiple unpaired t tests with FDR = 1%. For purposes of display, proteins present in WT MFGM but absent in Plin2-Null MFGM were assigned NSAF ratios of 1,000 (Log_2_ = 10). Proteins present in Plin2-Null MFGM but absent in WT MFGM were assigned NSAF ratios of 0.001 (Log_2_ = −10). Proteins with q-values ≤ 0.000001 are assigned values of 0.000001 (log_10_ = 6). WT enriched MFGM proteins are right and Plin2-Null enriched MFGM proteins are left of the vertical dotted line. Proteins found at LD-APM contact sites, BTN, XOR, Cidea and Plin2, are indicated by green diamond symbols. The horizontal line indicates q = 0.01. **(B)** STRING networks of proteins elevated (q > 0.01) in WT MFGM were identified using Cytoscape 3.9.1. Proteins forming a network with Plin2 include those implicated in formation of LD-APM contacts (XOR, BTN and Cidea) and Rab18 and FABP3. **(C)** Average ±SEM XOR:BTN and Cidea:BTN NSAF ratios in WT and Plin2-Null MFGM. Asterisk indicates *p* < 0.05, Students t-test. **(D)** Average ±SEM Rab18:BTN and Fabp3:BTN NSAF ratios in WT and Plin2-Null MFGM. Asterisk indicates *p* < 0.05, Students t-test. **(E)** STRING networks of proteins elevated (q < 0.01) in Plin2-Null MFGM are enriched in ER/chaperone and lipid metabolism proteins.

The formation of LD-APM contacts influences the protein composition of the membranes enveloping apocrine-secreted lipids (Monks, et al., 2016). To determine if the effects of Plin2 on the properties of LD-APM contacts are associated with altered membrane protein compositions we used untargeted LC-MS/MS analysis of the membrane fraction isolated from WT and Plin2-Null milk fat globules (MFG). Using a 5 peptide minimum and probabilities greater than 95% and 99% respectively for identifying peptides and proteins, we identified 226 and 341 proteins respectively in WT and Plin2-Null MFG membranes (MFGM) (Supplementary Table S1). Protein abundances were quantified using normalized spectral abundance factors (Zybailov, et al., 2006; Monks, et al., 2016). A volcano plot of the ratio of WT/Plin2-Null NSAF factor values versus false discovery rate adjusted *p*-values (q-values) of NSAF differences between WT and Plin2-Null MFGM proteins (Figure 3A) shows that 13 proteins were elevated (q < 0.01) on WT MFGM and 16 proteins were elevated (q < 0.01) on Plin2-Null MFGM. STRING analysis STRING (Search Tool for the Retrieval of Interacting Genes/Proteins) analysis was used to predict protein–protein interactions and functional interactions (Cytoscape 3.9.0) of proteins differentially elevated on WT MFGM (lower on Plin2-Null membranes). This analysis identifies 5 that are predicted to exist in a functional network with Plin2, including proteins hypothesized to mediate LD-APM interactions (BTN, XOR and Cidea), as well as ras-related protein-18 (Rab18) and fatty acid binding protein-3 (Fabp3) (Figure 3B). Elevated membrane levels of BTN, XOR and Cidea do not appear to be related to higher cellular levels of these proteins in WT dams. (Supplementary Figure S2) Significantly, BTN and XOR levels are elevated in mammary gland extracts from Plin2-Null compared to WT dams on L10, and cellular Cidea and Rab18 levels are not statistically different between WT and Plin2-Null dams (Supplementary Figure S2). We also find that WT MFGM have higher levels of folate receptor-1 (Fo1r, 264%) and lactadherin (Mfge8, 170%), whose MFGM abundances appear to be influenced by apocrine lipid secretion (Reinhardt and Lippolis 2008; Lu, et al., 2016), as well as modestly elevated levels of whey acidic protein (WAP, 121%). Elevated levels of BTN, XOR and Cidea in WT MFGM implicate Plin2 in organizing the protein complex proposed to mediate LD-APM contacts and possibly processes involved in their formation and/or stabilization.

Perilipin family members are widely associated with LD in different cell types, and potentially possess compensatory activities on LD cellular functions. Both Plin2 and perilipin-3 (Plin3) are expressed in the lactating mammary gland (Russell, et al., 2007). We did not detect Plin3 on WT MFGM, however we found small amounts on Plin2-Null MFGM, which correspond to 1/16th of Plin2 levels on WT MFGM (Supplementary Figure S1, Supplementary Table S1) and therefore are not consistent with a compensatory role in LD-APM interactions. Plin2 deletion also appeared to increase Plin3 on LD in sMEC. However, relative levels of Plin3 in mammary gland extracts were not significantly affected by Plin2 deletion (Supplementary Figure S1). Other perilipin family members were not detected on WT or Plin2-MFGM.

BTN is proposed to be the primary APM protein responsible for tethering LD to APM during apocrine lipid secretion (Mather, et al., 2019; Monks, et al., 2020). To further evaluate how Plin2 affects membrane proteins implicated in this process, we quantified NSAF ratios for proteins predicted to be functionally linked to Plin2 to those of BTN in MFGM from WT and Plin2-Null dams. The XOR:BTN NSAF ratio for WT MFGM was 1.8:1 (Figure 3C), which is consistent with the 2:1 ratio found previously by biochemical and image analysis of MFGM from multiple species (Mather and Keenan 1998; McManaman, et al., 2002; Monks, et al., 2016). We found a small decrease in the XOR:BTN ratio (1.6:1) on Plin2-Null MFGM that was not significant (Figure 3C). In contrast, the Cidea:BTN and Rab18:BTN NSAF ratios decreased significantly from ∼1:1 on WT MFGM to 0.5:1 on Plin2-Null MFGM (Figures 3C,D). The Fabp3:BTN NSAF ratio was also decreased by loss of Plin2, but not significantly (Figure 3D). Differences in the effect of Plin2 on these ratios suggest that it may influence MFGM protein levels by more than one mechanism, possibly through effects on APM and/or LD properties (Ozeki, et al., 2005), (Chong, et al., 2011) (Deng et al., 2021).

Proteins that were significantly elevated on Plin2-Null compared with WT MFGM corresponded primarily to the inter-related ER chaperone and fatty acid metabolism networks defined by STRING analysis (Figure 3E). Proteins in the ER chaperone network include thiol oxidases; protein disulfide isomerase-A1 (P4hb, 200%) and peroxiredoxin-4 (Prdx4, 210%); heat shock protein Hspa5 (173%) and calreticulin (Calr, 242%). Elevated enzymes in a fatty acid metabolism network included acetyl-CoA carboxylases-a (Acaca, 151%), acetyl-CoA synthetase-1 (Acsl1, 287%), fatty acid synthase (Fasn, 141%), ATP-citrate lyase (Acly, 135%). Elevation of proteins in these networks in Plin2-Null MFGM suggest that Plin2 may limit contributions of the ER and possibly other cellular sources of membrane to MFGM, and is consistent with proposed contributions of ER and secretory vesicles to these structures (Wu, et al., 2000; Honvo-Houeto, et al., 2016).

### 3.4 Contact zone molecular interactions

**FIGURE 4:**
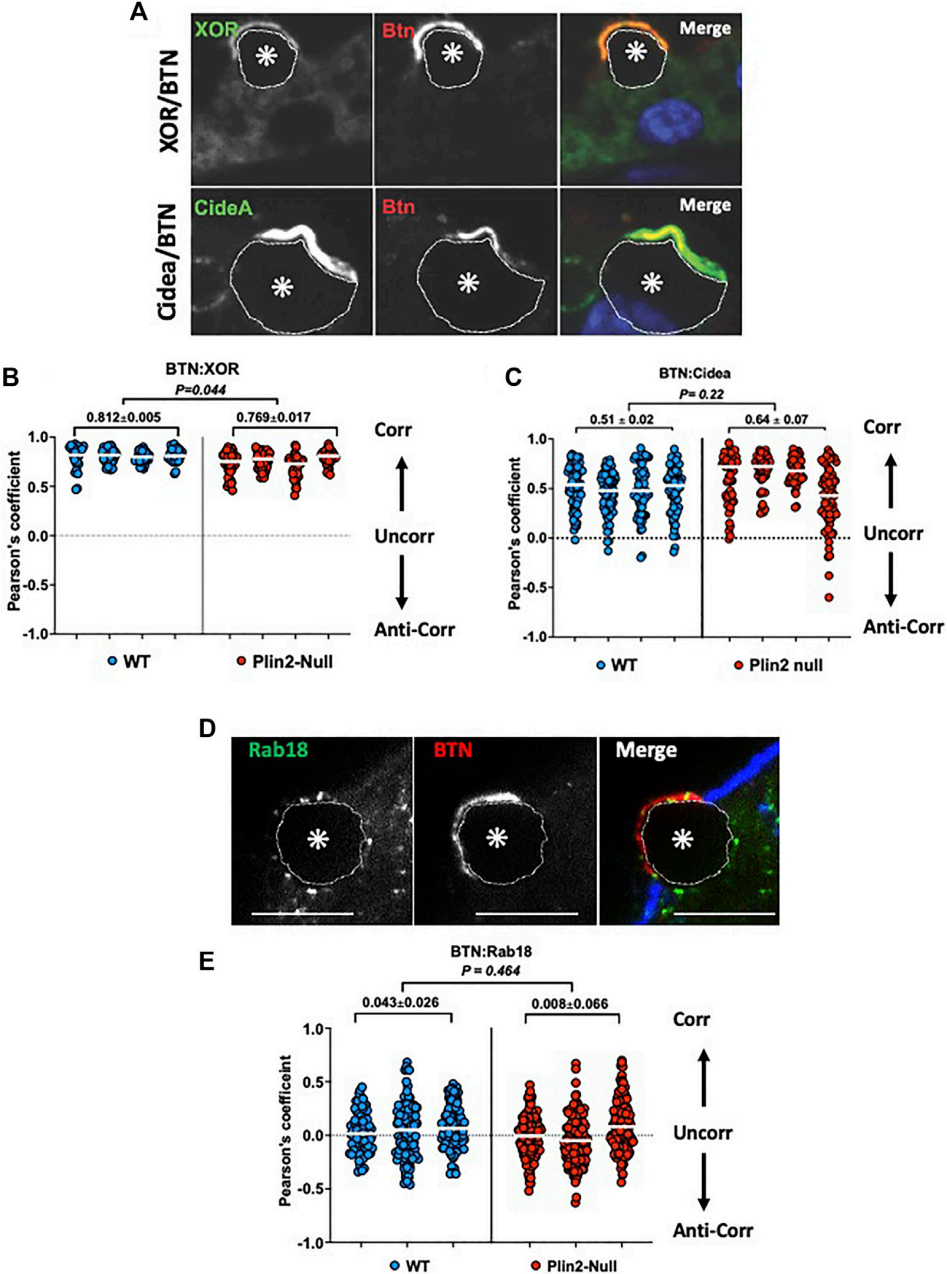
Molecular interactions at LD-APM contacts. Representative images of XOR (green/monochrome) or Cidea (green/monochrome) and BTN (red/monochrome) immunofluorescence **(A)** or Rab18 (green/monochrome) and BTN (red/monochrome) immunofluorescence **(D)** at LD-APM contacts in WT mammary glands following litter removal for 2 h at L10 to inhibit milk secretion. LD in images are indicated by asterisks and outlined by dotted white lines. Blue fluorescence in D is Alexa633-tagged wheatgerm agglutinin staining of the APM glycocalyx. Bar is 10 µ. **(B**,**C**,**E)** Pearson’s correlation coefficients describing immunofluorescence overlap between BTN and XOR **(B)**, BTN and Cidea **(C)**, or BTN and Rab18 **(E)** in 40–60 sMEC/animal from 3–4 WT and Plin2-Null dams following litter removal for 2 h at L10. Median values are indicated by white bars and average medians ±SEM are shown above each group. *p*-values for group differences were determined by nested *t*-test and are shown at the top of each figure. Pearson’s coefficients of 1, 0 and −1 respectively represent perfect positive correlation (Corr), no correlation (Uncorr.) and perfect negative correlation (Anti-corr.).

To directly assess Plin2 effects on the molecular interactions proposed to mediate LD-APM contacts, we quantified the extent of interaction between BTN, XOR, and Cidea at LD-APM contact sites in sMEC from WT and Plin2-Null dams at L10 following 2hrs of secretion inhibition. Confocal immunofluorescence (IF) imaging of WT sMEC, shows that XOR and Cidea immunofluorescence concentrate and overlap with that of BTN at LD-APM contact sites, although there appears to be some Cidea immunofluorescence that does not directly overlap with that of BTN (Figure 4A). In contrast, Rab18 immunofluorescence localizes in distinct domains at LD-APM contacts and on LD surfaces facing the cytoplasm (Figure 4A). We used Pearson’s correlation analysis to quantify the extent to which XOR, Cidea and Rab18 immunofluorescence overlaps with that of BTN at LD-APM contacts in WT and Plin2-Null cells (Figures 4B–D). Consistent with biochemical evidence that XOR and BTN physically interact in a macromolecular complex at LD-APM contact sites (Jeong, et al., 2009), we found that XOR:BTN immunofluorescence signals were highly correlated, with Pearson’s coefficients that approached the theoretical maximum of 1 (median Pearson’s coefficient = 0.831 ± 0.01) in WT cells (Figure 4B). XOR:BTN immunofluorescence in Plin2-Null cells were also highly correlated, however, there was a small but significant decrease the Pearson’s coefficient in Plin2-Null cells (median Pearson’s coefficient = 0.769 ± 0.034, *p* = 0.044), suggesting possible weakening of BTN-XOR interactions within the complex (Figure 4B). BTN and Cidea immunofluorescence signals at LD-APM contacts also were positively correlated in WT (median Pearson’s coefficient = 0.496 ± 0.020, N = 4) and Plin2-Null cells (median Pearson’s coefficient = 0.64 ± 0.07, N = 4) and median values were not significantly different (Figure 4D). Consistent with evidence from immunofluorescence images that Cidea localization at LD-APM contact sites extends beyond that defined by BTN:XOR interactions, we find that Pearson’s coefficients for Cidea:BTN immunofluorescence signals were not as positive (*p* < 0.001) and exhibited greater variation (*p* = 0.00175) than values for XOR:BTN in both WT and Plin2-Null cells. In contrast, median Pearson’s coefficients for Rab18:BTN immunofluorescence signals at LD-APM contacts in WT (0.043 ± 0.026) and Plin2-Null cells (0.008 ± 0.066) were close to 0 (Figure 4D) indicating that localization of Rab18 within LD-APM contact sites does not correlate with that of BTN in either WT or Plin2-Null cells and therefore is unlikely to be directly related to the formation of LD-APM contacts.

### 3.5 Glycocalyx remodeling

**FIGURE 5:**
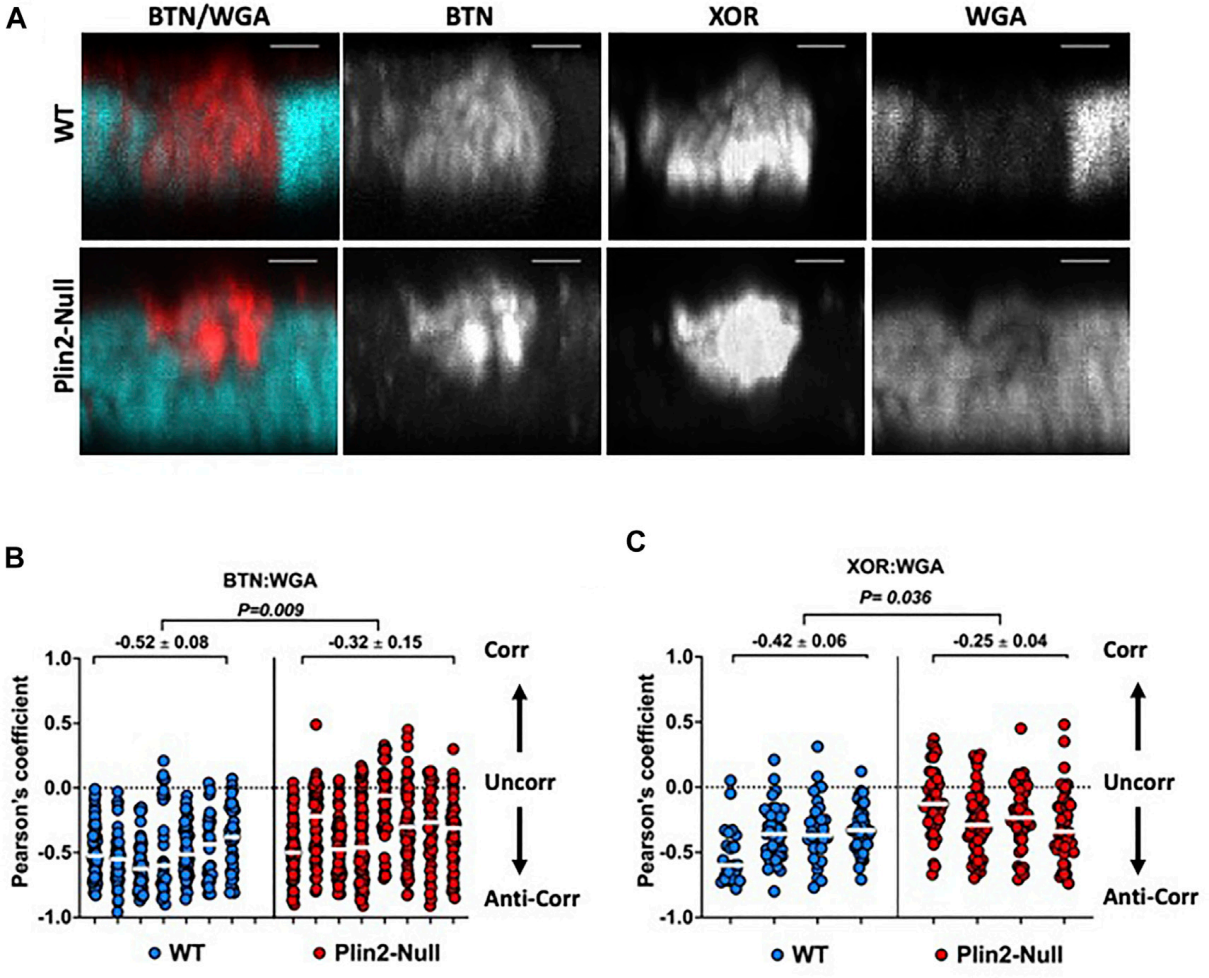
Glycocalyx remodeling at LD-APM contacts. **(A)** Representative 3D-projection images of mammary gland sections prepared from WT or Plin2-Null dams following removal of their litters for 2 h at L10 to inhibit milk secretion. The sections were immunostained with antibodies to BTN and XOR to identify LD-APM contacts and with Alexa633-tagged WGA (fl-WGA) to identify the glycocalyx. The images show exclusion of fl-WGA (blue green) from a BTN and XOR positive APM domain in WT sMEC, and partial exclusion of fl-WGA from a BTN and XOR positive APM domain in a Plin2 sMEC. Scale bars are 2 µm. **(B,C)** Pearson’s correlation coefficients describing the overlap between fl-WGA and BTN **(B)** or XOR **(C)** in 40–60 sMEC/animal from WT and Plin2-Null dams prepared as described in **(A)**. Median values are indicated by white bars and average medians ±SEM are shown above each group. *p*-values for group differences were determined by nested *t*-test and are shown at the top of each figure. Pearson’s coefficients of 1, 0 and −1 represent perfect correlation (Corr), no correlation (Uncorr.) and perfect negative correlation (Anti-corr.) respectively as shown in each figure.

Formation of LD-APM contacts remodels the sMEC glycocalyx, which results in exclusion of wheat germ agglutinin (WGA) positive substances from contact sites (Monks, et al., 2016). To determine if Plin2 actions contribute to this remodeling, we quantified the overlap of Alexa633-tagged WGA (fl-WGA) with BTN and XOR in sMEC from WT and Plin2-Null dams whose litters were removed for 2 h at L10 to inhibit secretion. Consistent with previous studies (Monks, et al., 2016), we found that BTN and XOR localized to, and were concentrated at, LD-APM contact sites lacking fl-WGA in WT cells (Figure 5A). In contrast, in Plin2-Null cells BTN and XOR appeared to localize within smaller areas of the membrane in which fl-WGA was not completely excluded (Figure 5A). When we quantified the overlap between BTN and XOR and WGA fluorescence signals by Pearson’s correlation in WT cells, we found marked negative correlations between BTN:WGA (−0.52 ± 0.08) and XOR:WGA (−0.42 ± 0.06) (Figures 5B,C). BTN and XOR immunofluorescence signals were also negatively correlated with fl-WGA in Plin2-Null dams (Figures 5B,C). However their median Pearson’s coefficients were significantly less negative (39–40%) than their WT values (BTN:WGA = −0.32 ± 0.15, *p* = 0.009; XOR:WGA = 0.24 ± 0.26, *p* = 0.036), which indicate that Plin2 promotes glycocalyx remodeling at LD-APM contact sites.

### 3.6 Secretion

**FIGURE 6:**
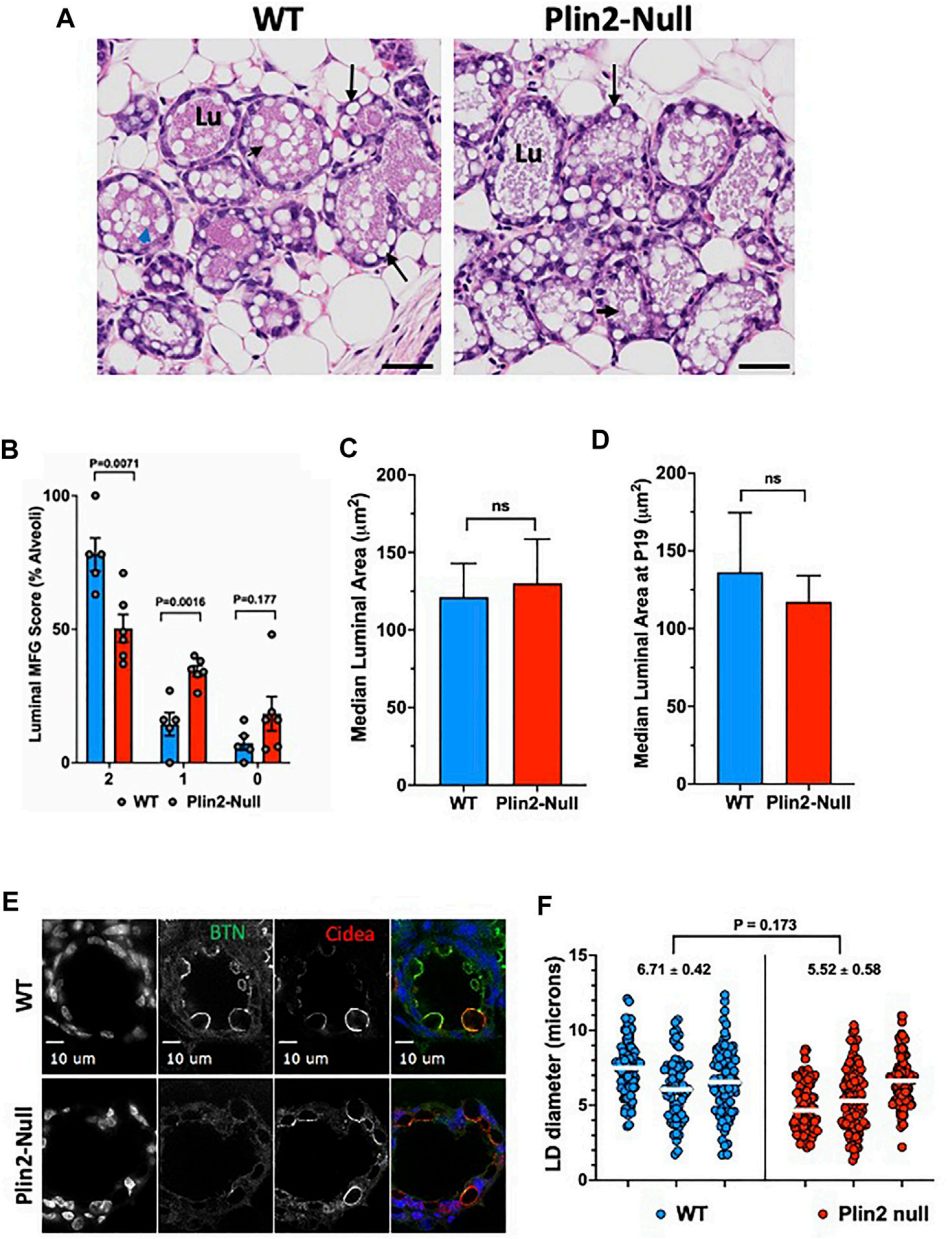
Apocrine lipid secretion in primiparous mice. **(A)** Representative histological images of mammary alveoli from primiparous WT and Plin2-Null mice on lactation day 1 (L1). Alveolar lumens (Lu) of WT mice are extensively filled with MFG (black arrow heads) the structures of secreted milk lipids. In contrast, LD appear to be retained in the epithelium (back arrow) and MFG are present in limited numbers in alveolar lumens of Plin2-Null dams. **(B)** Histological quantitation of luminal MFG. The data show the percentage of WT (N = 5, blue) and Plin2-Null (N = 6, red) mammary alveoli scored as having >3 MFG (score = 2); two or fewer (score = 1) or 0 (score = 0) MFG in their lumens at L1. *p* values were calculated by Students t-test from the results of 6 random sections per dam. **(C**,**D)** Histochemical quantitation of luminal areas in alveoli in sections from WT and Plin2-Null mammary gland at L1 **(C)** and P19 **(D)**. **(E)** Representative images of BTN (green/monochrome) and Cidea (red/monochrome) immunofluorescence in mammary glands of WT and Plin2-Null at P19. **(F)** Quantitation of LD diameters in mammary alveoli WT and Plin2-Null dams at P19. White bars are median values. Average median LD diameters are shown above each genotype. *p*-value of group differences was determined by nested t-test and is shown at the top of the figure.

Plin2-Null dams do not produce sufficient amounts of milk to support their first litters beyond the second day postpartum, but are able to support normal litter growth through weaning in subsequent pregnancies (McManaman, et al., 2013). Consistent with the ability of multiparous Plin2-Null dams to support normal litter growth, we find that the concentration of neutral lipids in milk of multiparous WT (0.78 ± 0.03 mmol/ml) and Plin2-Null (0.73 ± 0.03 mmol/ml; *p* = 0.34) dams on L10 is similar, and that there were no obvious differences in the number or physical properties of WT and Plin2 MFG (data not shown). The similarity of these values suggests that the effects of Plin2 absence on LD-APM contacts do not significantly impact apocrine secretion of lipids in lactating multiparous dams. To determine if the inability of Plin2-Null dams to support their first litters was associated with impaired lipid secretion, we quantified the number of MFG in lumens of mammary gland alveoli of primiparous WT and Plin2-Null dams on the first day of parturition [denoted as lactation day 1 (L1)] using a histological scoring system due to negligible quantities of milk at this time. At L1, most lumens in WT dams are filled with numerous MFG, whereas relatively few lumens from Plin2-Null dams have large numbers of MFG (Figure 6A). As shown in Figure 6B nearly 80% of lumens in WT mammary glands possessed multiple (>3) MFG (histological score = 2) whereas only 50% of lumens Plin2-Null mammary glands had multiple MFG (*p* = 0.007). In contrast, the percentage of lumens with only 1 or 2 MFG (histological score = 1) was 34% in Plin2-Null mammary glands compared to 14% in WT mammary glands (*p* = 0.0016). In addition, a greater percentage of Plin2-Null mammary glands (18%) had 0 MFG in their lumens (histological score = 0) compared to WT mammary galnds (7%) although the difference was not statistically significant (*p* = 0.177). These data indicate that in mice, Plin2 promotes the initiation of lipid secretion at the onset of lactation following their first pregnancy.

Previous studies of transgenic mice that express small amounts of an N-terminally truncated form of Plin2 found evidence of impaired alveolar maturation and reduction in LD size (Russell, et al., 2011). To control for the possibility that the effects of Plin2 deletion on the initiation of lipid secretion were related to developmental impairment of alveoli or LD size, we quantified luminal areas of alveoli in histological sections of mammary glands from WT and Plin2-Null dams just prior to parturition (pregnancy day 19) and on L1. We find that luminal areas are similar between WT and Plin2-Null dams at L1 and P19 (Figures 6C,D). We also quantified LD size in sMEC from sections of WT and Plin2-Null mammary glands on P19 after immunostaining for BTN and Cidea (Figure 6E). Although, there appeared to be more LD contacting the APM in WT sMEC than in Plin2-Null sMEC, we did not detect significant differences in LD diameter. These data indicate that effects of Plin2 on initiation of lipid secretion are unrelated to developmental effects on alveolar maturation or LD size.

## 4 Discussion

**FIGURE 7:**
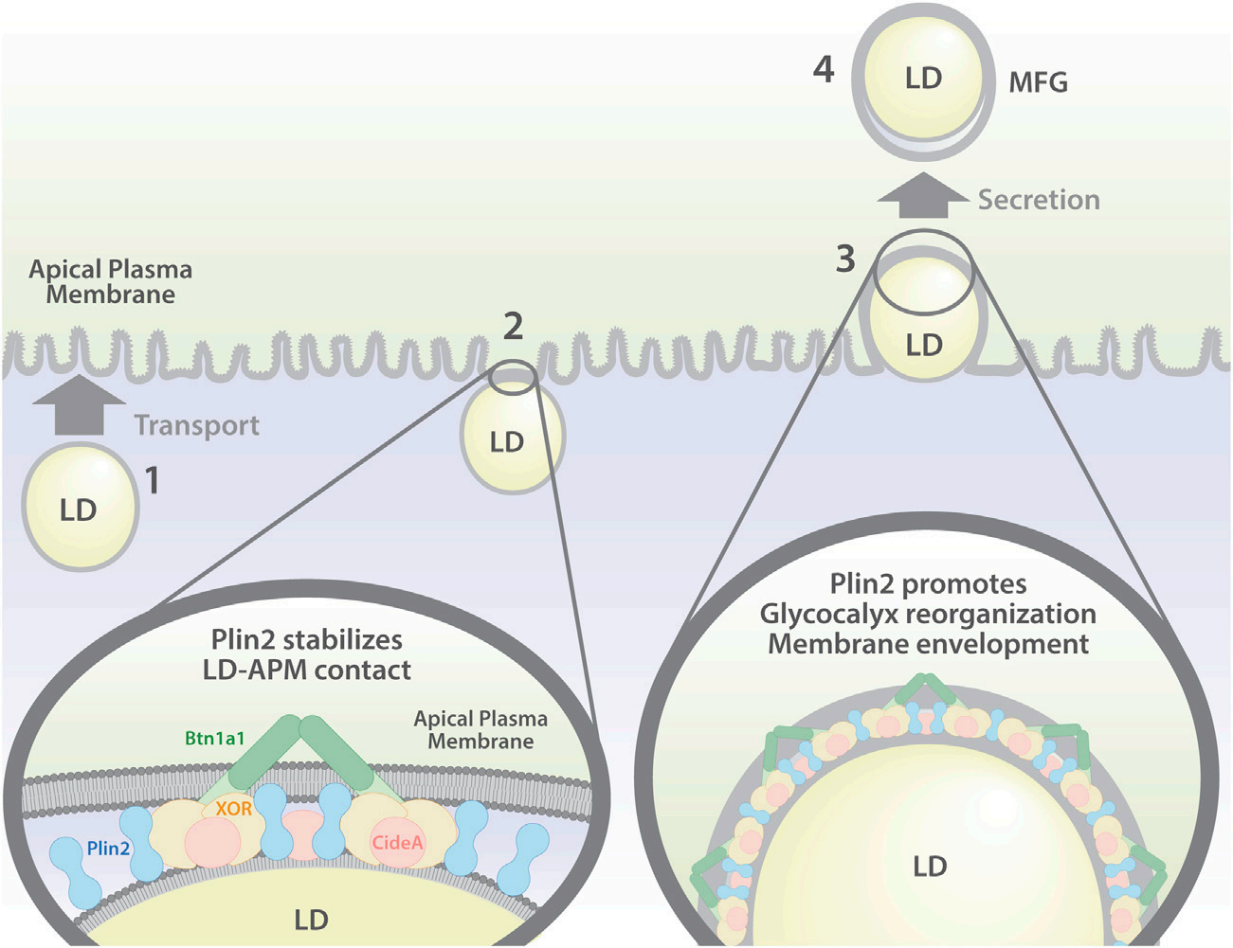
Model of LD-APM interactions and proposed roles of Plin2 in apocrine lipid secretion. Apocrine lipid secretion is proposed to involve four distinct steps: (1) LD transport to the APM; (2) formation of LD-APM contacts mediated by interactions between BTN and XOR (and possibly Cidea) and stabilized by Plin2; (3) Plin2 regulated envelopment of APM-bound LD and glycocalyx remodeling; (4) oxytocin dependent secretion of APM-enveloped LD to form milk fat globules (MFG).

Secretory epithelial cells of the mammary gland uniquely secrete LD by a unique apocrine mechanism that has long been understood to be integrally linked to formation and expansion of contacts between LD and the apical plasma membrane (Patton and Fowkes 1967; Mather and Keenan 1998; Wooding 2016; Mather, et al., 2019). However, there is considerable uncertainty about the mechanisms regulating these processes and their precise relation to lipid secretion (Jeong, et al., 2013; Monks, et al., 2016). Here we investigate the mechanisms regulating LD-APM interactions involved in apocrine lipid secretion. We demonstrate that in the mouse mammary gland, formation of LD-APM contacts and envelopment of membrane bound LD are independent processes, which are governed by distinct molecular interactions, and are independent of lipid secretion (Figure 7). We further show that Plin2, a constitutive LD protein previously proposed to tether LD to APM, enhances LD-APM stability and regulates LD envelopment by the APM, but is not required for formation of LD-APM contacts (Figure 7).

Stable contacts between LD and the APM are detected in sMEC of lactating animals (Wooding 1971) and multiple lines of evidence indicate that LD-APM contacts form in response to the activation of milk secretion (McManaman, et al., 2002). Intravital imaging of intact mammary glands of lactating mice revealed that apocrine secretion of LD depends on contraction of myoepithelial cells that surround glandular structures, induced by the pituitary hormone, oxytocin (Masedunskas, et al., 2017). Our discovery that LD continue to contact and become progressively enveloped by the APM in the absence of oxytocin-mediated LD secretion demonstrates that the processes controlling LD-APM interactions are distinct from those responsible for LD secretion. In addition, the demonstration that time courses describing differences in how the percentage of LD in contact with the APM, the size of APM-bound LD, and LD-APM envelopment respond to inhibtion of LD secretion support a multistep model of apocrine lipid secretion in which distinct cellular processes govern LD-APM interactions (Figure 7). The demonstation that LD-APM interactions do not require active LD secretion are consistent with previous demonstrations that LD can be secreted as membrane-enveloped structures in the absence of LD-APM contacts (Ogg, et al., 2004; Monks, et al., 2016), and further the concept that LD-APM interactions are likely to be specialized features of apocrine lipid secretion by sMEC to enhance the efficiency of lipid secretion (McManaman 2012; Monks, et al., 2016).

Ultrastructural evidence from several species document that LD-APM contacts are characterized by a 10–20 nm thick electron dense contact zone (Wooding 1971; Heid and Keenan 2005) that appears to be enriched in BTN, XOR and Plin2 (Mather and Keenan 1998; Mather, et al., 2019), which are proposed to form a multimeric complex (McManaman, et al., 2002; Jeong, et al., 2013) that tethers LD to the APM (Mather, et al., 2019; Monks, et al., 2020). Gene deletion studies have demonstrated that BTN and XOR are required for formation of stable LD-APM contacts (Ogg, et al., 2004; Monks, et al., 2016). In contrast, our finding that Plin2 is not required for formation of close contacts between LD and the APM or for concentrating BTN and XOR at LD-APM contact sites, shows that it is not essential for tethering LD to APM, as previously proposed. Rather, the demonstration that Plin2 significantly increases the percentage of LD that contact the APM and the area of contact between LD the APM, indicates it has previously undescribed functions in stabilizing LD-APM contacts and enhancing LD-APM interactions that mediate LD membrane envelopment (Figure 7). We acknowledge that these conclusions are drawn from analyses of static images and that dynamic approaches such as intravital-imaging (Masudenskas et al., 2017; Mather et al., 2019) of LD growth, transport, envelopment by APM and oxytocin-release into the milk will be required to fully define how Plin2 affects specific processes of apocrine secretion.

The mechanisms responsible for the effects of Plin2 LD-APM interactions remain uncertain and may be complex, involving direct and indirect actions. Contact between LD and the APM is associated with APM remodeling (Peixoto de Menezes and Pinto da Silva 1978; Mather and Keenan 1998; Monks, et al., 2016) and re-localization and concentration of BTN, XOR and Cidea at LD-APM contact sites (McManaman, et al., 2002; Jeong, et al., 2013; Monks, et al., 2016). Studies demonstrating that these membrane responses are largely prevented when formation of LD-APM contacts are inhibited by deleting XOR (Monks, et al., 2016) provide direct evidence of their dependence on the molecular processes that mediate LD-APM interactions. Our finding that, in conjunction with reducing the size of LD-APM contacts, Plin2 deletion interferes with glycocalyx remodeling at LD-APM contacts and decreases the levels of BTN, XOR and Cidea on MFGM, thus suggest that Plin2 acts, in part, by influencing the molecular interactions that regulate the organization and/or stability of LD-APM contacts. Prior studies showing that Plin2 co-localizes with BTN and XOR at LD-APM contact sites and is isolated as a complex with these proteins from MFGM (McManaman, et al., 2002) suggest that Plin2 may directly affect interactions between BTN and XOR that mediate LD-APM contacts. However, we find that Plin2 deletion has only a small effect on BTN and XOR interactions at sites of LD-APM contact. Alternatively, the demonstration that the C-terminal 4-helix bundle domain of Plin2 binds to bind membrane bilayers directly (Chong, et al., 2011), has led to the hypothesis that it may indirectly affect LD-APM contacts through interactions with plasma membrane lipids (McManaman 2012). Additionally, Plin2 potentially influences LD-APM interactions through effects on LD properties. Plin2 knockdown has been shown to enhance contact between LD and endoplasmic reticulum membranes through a mechanism involving increased LD Rab18 (Ozeki, et al., 2005). We find that Plin2 effects on LD-APM interactions are associated with elevated Rab18 levels on MFGM. However, Rab18 did not specifically localize to LD-APM contact sites and its localization was unrelated to that of BTN, which suggest that it does not directly mediate LD-APM contacts. Nevertheless, Rab18 has multiple functions including regulation of secretion, vesicle trafficking and LD dynamics (Deng et al., 2021) that potentially enhance the stability of LD-APM interactions. LD size is also a possible regulator of LD-APM stability through enlargement of the area of contact between membrane surfaces. Consistent with this possibility, we find a significant increase in the size of LD contacting the APM in conjunction with the expansion of the LD-APM contact zone in WT sMEC. However, there is a comparable increase in size of LD contacting the APM in the absence of significant expansion of the LD-APM contact zone in Plin2-Null cells. Thus the effects of Plin2 on the apparent stability of LD-APM contacts do not appear to be directly related to LD size, and the mechanisms responsible for regulating LD size at LD-APM contacts appear to be distinct from those regulating the area of contact between LD and APM membranes. Additional immuno-EM and/or super-resolution imaging of the macromolecular complexes that stabilize LD-APM contact will yield many more insights into their formation.

Significantly, alterations in LD-APM contacts induced by Plin2 loss appear to be associated with reduced LD secretion and possibly to impaired lactation (McManaman, et al., 2013) at the onset of lactation (L1) in primiparous dams. These results are consistent with evidence from previous studies showing that preventing formation of LD-APM contacts by deleting BTN or XOR impairs apocrine lipid secretion and prevents or delays in lactation (Vorbach, et al., 2002; Ogg, et al., 2004; Monks, et al., 2016). Together, data from these studies support the proposal that LD-APM interactions enhance the efficiency of apocrine lipid secretion (McManaman 2012; Monks, et al., 2016), which appears to be an important determinant of initial lactation success. LD that are not attached to the APM may also be secreted less-efficiently and remain within the cell following oxytocin stimulated secretion. Again, quantitative, intravital imaging of large numbers of LD is necessary to rigorously test this hypothesis. Nevertheless, data from this study and previous BTN, XOR, and Plin2 deletion studies demonstrate that apocrine lipid secretion is not strictly dependent on LD-APM interactions. We propose that LD-APM interactions in sMEC may represent an evolutionary gain of function adaptation to facilitate lipid delivery to neonates.

## Data Availability

The datasets presented in this study can be found in online repositories. The names of the repository/repositories and accession number(s) can be found below: The mass spectrometry proteomics data have been deposited to the ProteomeXchange Consortium *via* the PRIDE [1] partner repository with the dataset identifier PXD035020 and 10.6019/PXD035020.
