# Supplementary figures and images for: Perilipin-2 promotes lipid droplet-plasma membrane interactions that facilitate apocrine lipid secretion in secretory epithelial cells of the mouse mammary gland
Source: Front Cell Dev Biol. 2022 Sep 9;10:958566. doi: 10.3389/fcell.2022.958566 (PMC9500548; doi:10.3389/fcell.2022.958566)

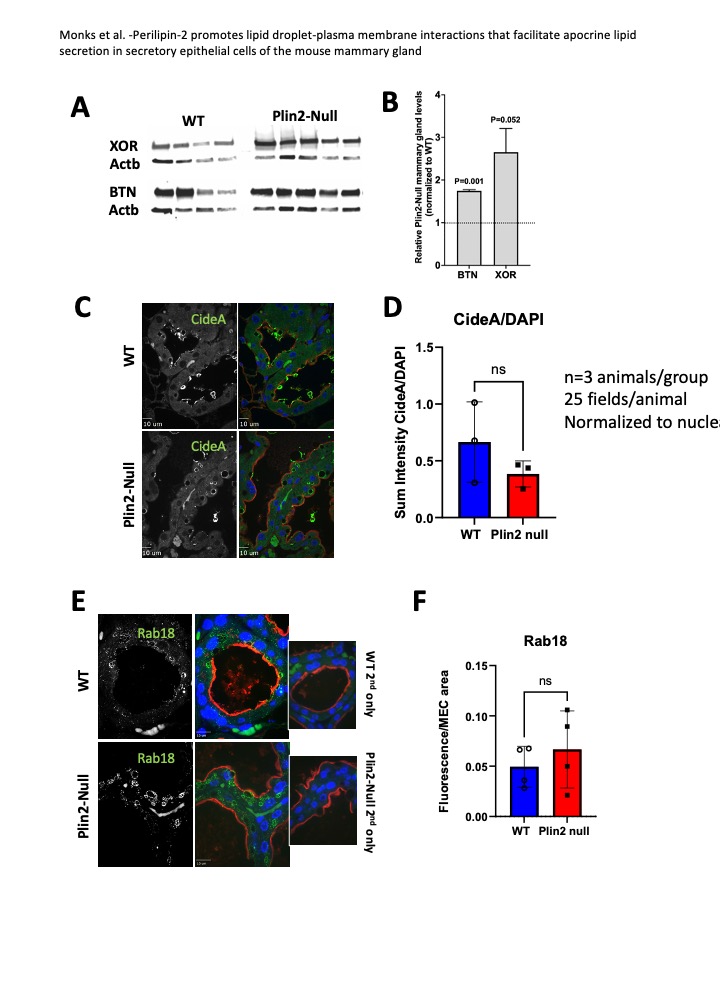

Supplement: Supplementary file 1 [file Image2.JPEG]

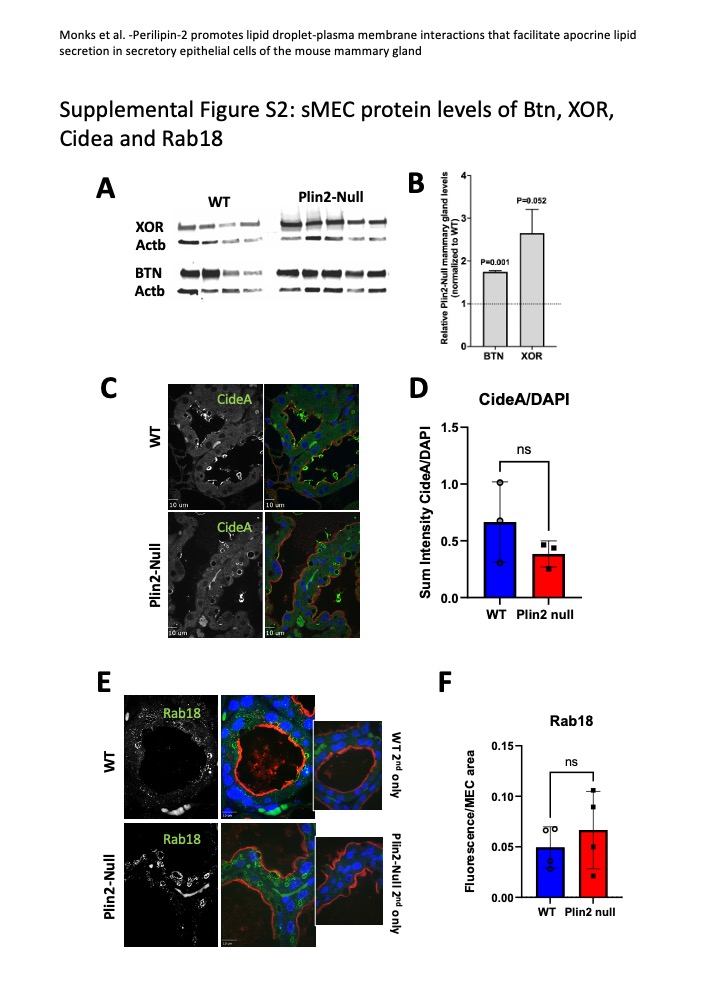

Supplement: Supplementary file 3 [file Image1.jpg]
