# Supplementary material for: Perilipin-2 promotes lipid droplet-plasma membrane interactions that facilitate apocrine lipid secretion in secretory epithelial cells of the mouse mammary gland
Source: Front Cell Dev Biol. 2022 Sep 9;10:958566. doi: 10.3389/fcell.2022.958566 (PMC9500548; doi:10.3389/fcell.2022.958566)
Supplement: Supplementary file 4 [file DataSheet1.docx]

Supplemental Figure legends

Figure 1. Plin3 localization and quantitation in WT and Plin2-Null mammary glands. (A) Representative confocal fluorescence images of WT and Plin2-Null mammary glands immunostained with primary antibodies to Plin2 and Plin3 and Alexa-488 (Plin2, green/monochrome) and -596 (Plin3, red/monochrome) labeled secondary antibodies. Nuclei were identified by DAPI stains. Secondary antibody only images are shown for comparison. Note the markedly elevated levels of Plin3 on LD in sMEC from Plin2-Null glands. (B) Representative immunoblots of Plin2, Plin3 and β-actin (Actb) in extracts of WT and Plin2-Null mammary glands. (C) Immunoblot quantitation of mammary gland levels of Plin2 and Plin3 levels normalized to Actb). (D) Quantitation of Plin2 and Plin3 on WT and Plin2-Null MFGM by LC/MS/MS using normalized spectral abundance factors for quantitation. WT MFGM have robust amounts of Plin2 but undetectable amounts of Plin3, whereas Plin2-Null MFGM have no Plin2 and small amounts of Plin3. (E) Confocal immunofluorescence images of Plin2-Null mammary glands immunostained for Plin3 and Cidea, showing patchy localization of Plin3 distributed on the surface of LD in contact with the APM whereas Cidea localizes at the LD-APM interface.

Figure 2. Quantitation of BTN, XOR, Cidea and Rab18 in sMEC of WT and Plin2-Null mammary glands. (A) Representative immunoblots of BTN, XOR and β-actin (Actb) in mammary gland extracts from WT and Plin2-Null dams at L10. (B) Quantitation of BTN and XOR in extracts of WT and Plin2-Null mammary glands at L10. Values are average amounts of BTN and XOR in extracts of Plin2-Null mammary glands normalized to levels in WT mammary gland extracts from two experiments. P values were determined by Students t-test and are shown above respective BTN and XOR averages. (C) Representative confocal fluorescence images of Cidea (green), Alexa-594 labeled wheatgerm agglutinin and DAPI stained nuclei (blue) in mammary gland sections from WT and Plin2-Null dams at L10. (D) Quantitation of Cidea immunofluorescence intensity in mammary gland sections. Values are average sum intensities of Cidea immunofluorescence normalized to DAPI fluorescence in 25 fields/animal for 3 WT and Plin2-Null dams. (E) Representative confocal fluorescence images of Rab18 (green), Alexa-594 labeled wheatgerm agglutinin (Red) and DAPI stained nuclei (blue) in mammary gland sections from WT and Plin2-Null dams at L10. Corresponding images of sections stained with secondary antibodies only are shown to demonstrate primary antibody staining specificity. (F) Quantitation of Rab18 immunofluorescence intensity in mammary gland sections. Values are average sum intensities of Rab18 immunofluorescence normalized to sMEC area in 5 random images per mouse mammary gland in 4 WT and Plin2-Null dams.
